# The hematopoietic compartment is sufficient for lupus development resulting from the *POLB-Y265C* mutation

**DOI:** 10.1371/journal.pone.0267913

**Published:** 2022-04-29

**Authors:** Tania Rahim, Madison A. Levinson, Kelly E. W. Carufe, Matthew Burak, Rithy Meas, Stephen Maher, Alfred L. M. Bothwell, Naomi Gades, Joann B. Sweasy

**Affiliations:** 1 Department of Therapeutic Radiology, Yale University School of Medicine, New Haven, CT, United States of America; 2 Department of Cellular and Molecular Medicine and University of Arizona Cancer Center, Tucson, AZ, United States of America; 3 Department of Genetics, Yale University School of Medicine, New Haven, CT, United States of America; 4 Moderna Therapeutics, Cambridge, MA, United States of America; 5 Department of Immunology, Yale University School of Medicine, New Haven, CT, United States of America; 6 Department of Comparative Medicine, Mayo Clinic Arizona, Scottsdale, AZ, United States of America; Universite Paris-Saclay, FRANCE

## Abstract

Systemic lupus erythematosus is a chronic disease characterized by autoantibodies, renal and cutaneous disease, and immune complex formation. Emerging evidence suggests that aberrant DNA repair is an underlying mechanism of lupus development. We previously showed that the *POLB*^Y265C/C^ mutation, which results in development of an aberrant immune repertoire, leads to lupus-like disease in mice. To address whether the hematopoietic compartment is sufficient for lupus development, we transplanted bone marrow cells from *POLB*^Y265C/C^ and *POLB*^+/+^ into wild-type congenic mice. Only mice transplanted with the *POLB*^Y265C/C^ bone marrow develop high levels of antinuclear antibodies and renal disease. In conclusion, we show that the hematopoietic compartment harvested from the *POLB*^Y265C/C^ mice is sufficient for development of autoimmune disease.

## Introduction

Systemic lupus erythematosus (SLE or lupus) is an autoimmune disease that affects over 5 million people worldwide. Monozygotic twin studies suggest that lupus results from genetic predisposition in combination with environmental exposures, including ultraviolet light (for a review see [1]). Common risk alleles associated with lupus are in genes associated with immune system function, as would be expected for an autoimmune disease. Diagnostic criteria associated with lupus include the presence of high titers of autoantibodies including antinuclear antibodies, malar rash, photosensitivity, renal disorder, non-erosive arthritis, and pleuritis or pericarditis [2].

Recent evidence indicates that aberrant DNA repair is associated with lupus (for a review see [1]). Previously we discovered that a genetic variant of the *POL B* gene, which encodes the DNA polymerase β protein, induced lupus development in a mouse model [3]. The *POLB*^Y265C/C^ mice expressing the Pol β-Y265C genetic variant develop high levels of antinuclear antibodies (ANA), glomerulonephritis with immune complex formation, and lupus-like skin lesions [3]. In addition, VDJ recombination in the *POLB*^Y265C/C^ mice is aberrant in that the complementarity determining region 3 (CDR3) of the immunoglobulin heavy chain (IgH) is significantly shorter than what is observed in wild-type (WT) controls, likely due to aberrant end-joining. The frequency of somatic hypermutation is also elevated in the *POLB*^Y265C/C^ mice.

DNA polymerase β (Pol β) functions is known to be a key DNA polymerase during base excision repair and recently it was shown to function in alternative end-joining [4, 5]. Both of these DNA repair pathways function during development of the immune repertoire. The base excision repair (BER) pathway is responsible for the processing of 20,000–30,000 lesions per cell per day [6]. These lesions arise due to the presence of reactive oxygen and nitrogen species (RONs) in cells. Monofunctional DNA glycosylases recognize and remove oxidized and methylated bases, leaving an abasic site. Apurinic/apyrimidinic exonuclease 1 (APE1) cuts the DNA 5’ to the abasic site, leaving a 3’OH and 5’deoxyribose phosphate (5’dRP) group. Pol β removes the dRP group and fills in the single nucleotide gap followed by sealing of the nick by the DNA ligase 3α (Lig3α)-X-ray Cross-Complimenting 1 (XRCC1) complex (for a review see [5]). Bifunctional DNA glycosylases remove the damaged base and cut the DNA backbone, leaving DNA ends that must be remodeled by enzymes including APE1 and polynucleotide kinase to generate a 3’OH and 5’phosphate. Pol β then fills in cα-XRCC1 seals the nick. During alternative end-joining, Pol β fills short gaps in DNA prior to ligation of the nick [4].

The Pol β-Y265C variant was identified by our group in a genetic screen for mutator variants of this enzyme [7]. Later, it was identified in a gastric carcinoma [8]. Pol β-Y265C exhibits low catalytic activity and strong mutator activity *in vitro* [7, 9]. Cells expressing the Pol β-Y265C variant also exhibit an increased mutation frequency, including a significant increase in microhomology-mediated deletions [10]. Interestingly, previous work indicated that a single nucleotide polymorphism that is correlated with reduced expression of Pol β, which could be analogous to low catalytic activity, is linked to lupus development [11, 12].

Previous work using other mouse models has shown that the hematopoietic compartment is sufficient for the development of autoimmune diseases including lupus and diabetes. Specifically, transplantation of bone marrow cells from mice with these diseases leads to development of autoimmune phenotypes in non-disease-prone recipient mice [13, 14]. To determine if the hematopoietic compartment is sufficient for development of lupus, we transplanted bone marrow cells isolated either from the *POLB*^Y265C/C^ or *POLB*^+/+^ mice into “Pep Boy” congenic recipients. We found that mice engrafted with bone marrow cells harvested from the *POLB*^Y265C/C^ mice developed high levels of ANA and renal disease. Significant lupus symptoms were not observed in mice engrafted with bone marrow harvested from the *POLB*^+/+^ mice. These results suggest that expression of Y265C-Pol β in hematopoietic cells is sufficient for development of lupus.

## Methods

### Bone marrow transplantation

Bone marrow was harvested from either *POLB*^Y265C/C^ or *POLB*^Y265+/+^ littermates at 4–6 weeks of age and 2–5 million cells were retro-orbitally injected into lethally irradiated Pep Boy recipient mice. Pep Boy mice are a C57Bl/6 congenic strain that carry the pan differential leukocyte marker *Ptprc*, which is commonly known as CD45.1. The *POLB*^Y265C/C^ and *POLB*^+/+^ mice are on an F1 C57Bl/6 x S129/SVJ background and so have the leukocyte marker CD45.2, making it possible to monitor engraftment of donor bone marrow in the recipients. Engraftment was monitored by FACs analysis of live cells from a sample of blood with using antibodies raised against CD45.1 (FITC-Tonbo Sciences) and CD45.2 (APC-Tonbo Sciences) six weeks after transplantation. All experiments were conducted with approval from the Yale University IACUC Committee. The method of euthanasia was carbon monoxide fixation. Isoflurane was used for anesthesia for retro-orbital blood collection and administration of bone marrow. Minimization of pain and suffering was by euthanasia.

### Anti-nuclear antibodies (ANA)

ANA was measured by coating human HEp-2 cells fixed slides (Diasorin) with mouse serum using an Alexa 488® goat-anti-mouse IgG secondary antibody (Invitrogen) as per manufacturer’s instructions. Coated slides were mounted with ProLong Gold anti-fade reagent (Invitrogen) and digitally photographed with a Nikon EVOS fluorescence microscope. Pixel Fluorescent intensity was measured by ImageJ® software.

### Scoring of kidney pathology

Kidneys from the mice were preserved and stained with hematoxylin and eosin (H and E) as previously described [3]. The severity of kidney lesions was assessed for mesangial matrix thickening, increased mesangial cellularity, glomerular enlargement, segmental tuft atrophy, glomerular crescents, Bowman’s capsular fibrosis, glomerulosclerosis, perivascular round cell infiltration, tubular epithelial proliferation, tubular epithelial atrophy, and tubular dilation with cellular casts. All changes were graded semi-quantitatively using a scoring scale in which no features are observed is a score of 0; Mild; notable feature not disrupting pre-existing tissue or limited to small area is a score of 1; moderate response with a notable feature disrupting existing tissue, but limited to a small area is a score of 2; and a marked, severe response with a notable feature replacing pre-existing tissue over a large area is a score of 3. For each sample, scores were summed to obtain a final score.

#### Flow Cytometry Analyses (FACs)

To quantify numbers of plasma cells, FACs analysis using antibodies raised against CD19 (PE-BD Biosciences) and CD138 (APC, Biolegend) was performed on live cells of blood samples taken from the mice. Activated germinal center B cells from spleens were quantified as CD19^+^IgD low GL7^+^CD95^+^ (PE, BD Biosciences; APC, eBioscience; AF488, eBioscience; PeCy7, BD Biosciences) as we previously described [3]. Follicular helper T cells from spleens were quantified as CD4^+^, CD44 low, PD1^+^, CXCR5^+^ (Pacific Blue, Biolegend; APC, BD Biosciences; FITC, eBioscience; PE, eBioscience) as previously described [3].

#### Immunohistochemistry of germinal centers

Staining was performed as previously described [3].

#### Statistical analysis

Graphpad Prism was employed for statistical analysis of the data.

## Results and discussion

The lupus-prone *POLB*^Y265C/C^ mice develop high levels of ANA, glomerulonephritis, and lupus-like skin lesions. Lupus-like disease in these mice could arise as a result of a defect in hematopoietic cells such as aberrant development of the immune repertoire. Another mechanism underlying disease development in our mice could be related to aberrant BER within the target organs, including kidneys and skin, resulting in dying cells liberating antigenic cellular debris. To determine if hematopoietic cells from the *POLB*^Y265C/C^ mice are sufficient for lupus development, we harvested bone marrow from either the *POLB*^Y265C/C^ or *POLB*^+/+^ mice and transplanted it into “Pep Boy” recipients as described in methods. The use of Pep Boy mice makes it possible to monitor engraftment of the bone marrow cells from the *POLB*^Y265C/C^ and *POLB*^+/+^ mice in the Pep Boy mice and to determine if their presence is associated with the manifestations of lupus.

### Mice reconstituted with bone marrow cells from the *POLB*^Y265C/C^ mice develop high levels of ANA

**Fig 1:**
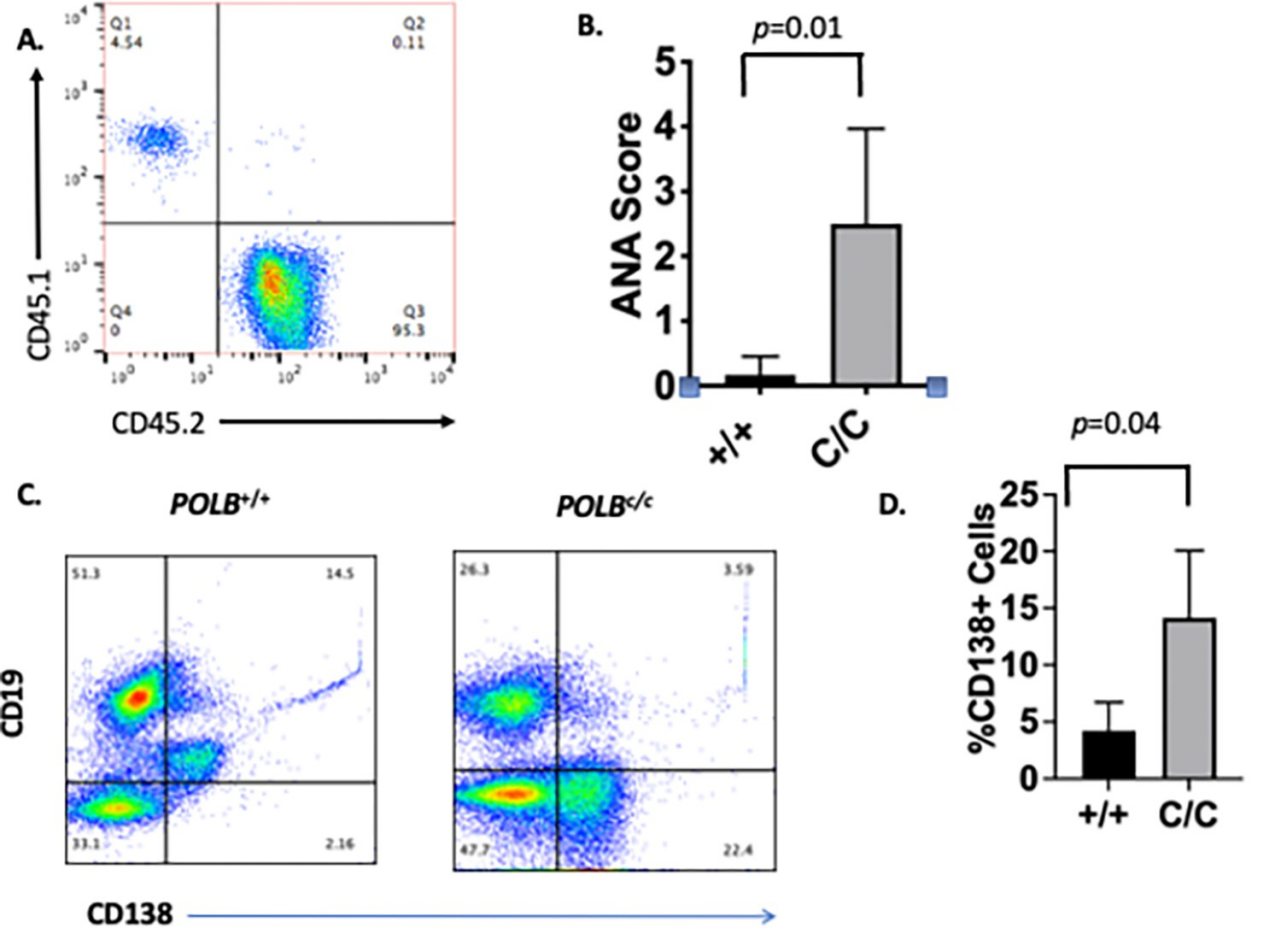
Mice engrafted with bone marrow from the *POLB*^Y265C/C^ mice develop high levels of antinuclear antibodies. A. Representative example of a FACs plot demonstrating successful engraftment of bone marrow cells in the Pep Boy mice from the *POLB*^Y265C/C^ donor. Similar results were observed when the *POLB*^+/+^ mice served as donors. B. ANA scores of sera from *POLB*^+/+^ and *POLB*^C/C^ mice. C. Representative example of a FACs plot showing increased levels of CD138^+^ plasma cells isolated from the blood of the *POLB*^+/+^ and *POLB*^C/C^ mice. D. Quantification of CD138^+^ cells. T-tests were used to determine if the means of each of the two groups were significantly different from each other.

Bone marrow cells were harvested from 4–6 week-old *POLB*^Y265C/C^ or *POLB*^+/+^ mice as described in methods and injected retro-orbitally into Pep Boy recipients that had been lethality irradiated (see methods). Six weeks after injection of the bone marrow cells, blood from the mice was analyzed for reconstitution as described in methods. Between 86–99% of the CD45 cells isolated from the recipients were marked with CD45.2, an example of which is displayed in Fig 1A, suggesting that significant reconstitution of the bone marrow of the Pep Boy recipients had taken place with bone marrow cells isolated from either the *POLB*^Y265C/C^ or *POLB*^+/+^ mice.

Approximately 9–12 months later, all recipients were euthanized and underwent a complete necropsy. Serum from the mice was analyzed for the presence of ANA. Recipients of bone marrow cells from the *POLB*^Y265C/C^ mice exhibited significantly (*p = 0*.*026*) elevated levels of ANA compared to recipients of bone marrow cells from the *POLB*^+/+^ mice (Fig 1B). Notably, the levels of ANA are similar to what is observed in *POLB*^Y265C/C^ mice at ages 6–12 months [3]. Recipients of bone marrow from the *POLB*^Y265C/C^ mice have significantly increased levels of CD138^+^ plasma cells in their blood versus controls, as would be expected given their high levels of ANA (Fig 1C). This result suggests that hematopoietic cells from the *POLB*^Y265C/C^ mice are sufficient for the production of ANA in the recipient mice.

The high levels of ANA observed in mice engrafted with bone marrow from the *POLB*^Y265C/C^ mice are likely a result of the development of an aberrant immune repertoire. Both VDJ recombination and somatic hypermutation are altered in the *POLB*^Y265C/C^ mice [3]. Specifically, the CDR3 regions of the immunoglobulin heavy chains are significantly shorter in these mice than what is observed in their wild-type siblings. Subsequent work showed that Pol β functions in VDJ recombination [4]. We suspect that the low catalytic activity of the Pol β-Y265C protein results in defective gap-filling during microhomology-mediated end-joining and that this leads to the short CDR3 regions we observe in *POLB*^Y265C/C^ mice. The deficient gap-filling activity of Pol β-Y265C protein is also likely contributing to the increased somatic hypermutation frequency observed in these mice. Therefore, it is likely that the aberrant VDJ recombination and somatic hypermutation in B cells is an underlying mechanism leading to the production of ANA in the recipients engrafted with bone marrow from the *POLB*^Y265C/C^ mice.

### PEP Boys reconstituted with bone marrow from *POLB*^Y265C/C^ mice develop renal disease

**Fig 2:**
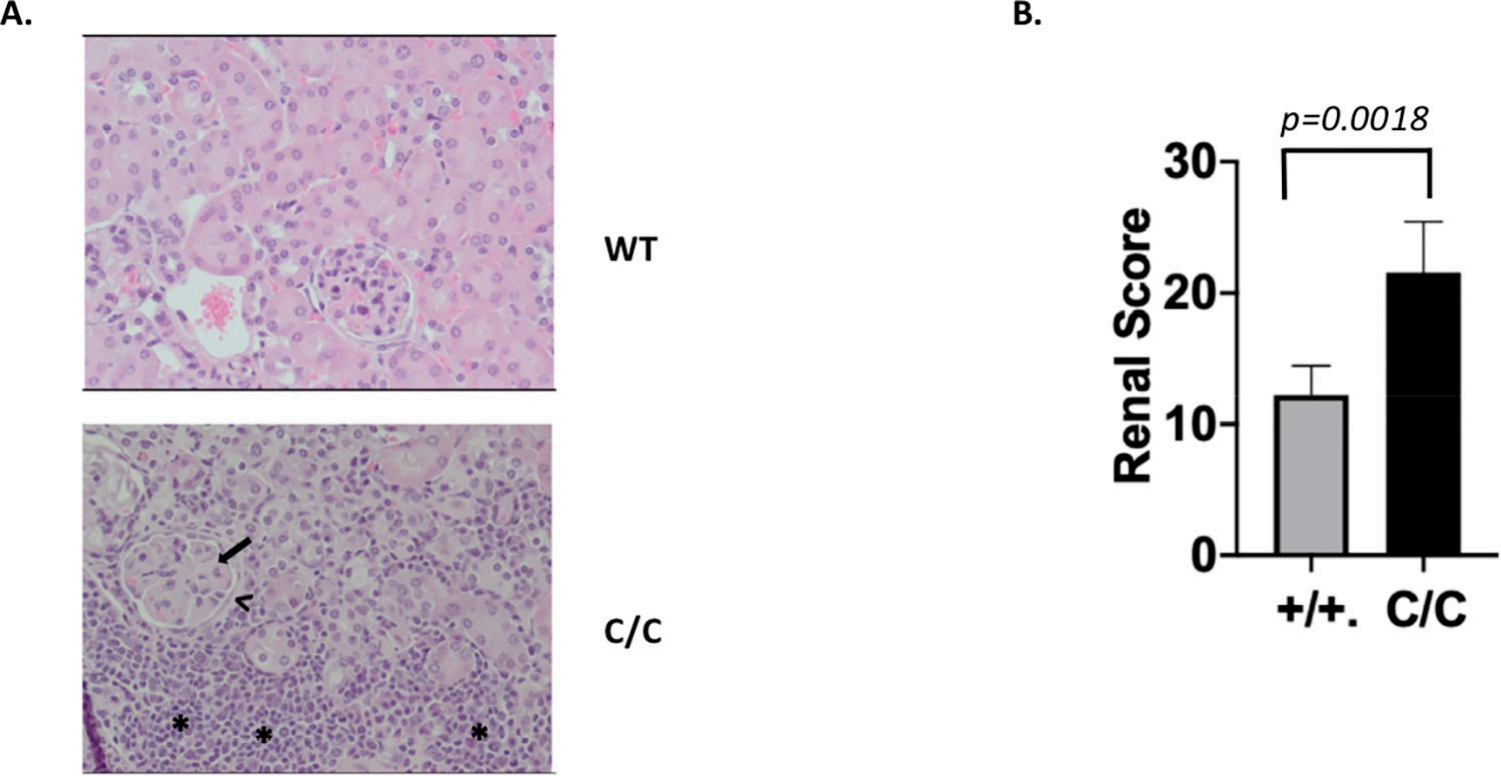
Severe renal disease in mice engrafted with bone marrow from the *POLB*^Y265C/C^ mice. A. Representative examples of H and E sections with the following features: interstitial inflammation with infiltrates of lymphocytes and plasma cells obliterating the renal tubulointerstitium (*asterisk*); generalized hyaline thickening of glomerular capillary basement membrane (*arrow*); thickened Bowman’s capsule (*arrowhead*). B. Several features of renal disease were scored as described in Methods and added together to obtain the final scores.

A hallmark phenotype of lupus-prone mouse models is the development of renal disease and the *POLB*^Y265C/C^ mice develop severe renal disease by 12 months of age [3]. H and E sections of the kidneys of bone marrow recipient mice were scored by a pathologist using the scoring system described in methods. We found that transplantation of bone marrow cells from the *POLB*^Y265C/C^ mice resulted in renal disease in the Pep Boy recipients, as signified by higher average renal pathology score of 21.6±3.9 (n = 7) for recipients receiving bone marrow from the *POLB*^Y265C/C^ mice versus an average score of 12.3±2.2 (n = 4) for recipients of bone marrow from *POLB*^+/+^ mice (Fig 2A). Immune complex formation in the kidneys is indicative of the severity and mechanism of renal disease. Immune complex formation is thought to result from either circulating immune complexes or planted antigen. Only one of the seven mice transplanted with bone marrow harvested from the *POLB*^Y265C/C^ mice exhibited immune complex formation in the kidneys. No immune complex formation was observed in the kidneys from Pep Boys transplanted with bone marrow from the *POLB*^+/+^ mice. These results suggest that hematopoietic cells from the *POLB*^Y265C/C^ mice are sufficient for the development of renal disease, but not for immune complex formation.

Renal pathology is of greater severity in the recipients of bone marrow from the *POLB*^Y265C/C^ compared to *POLB*^+/+^ mice. However, only one of the seven recipients of the *POLB*-Y265C bone marrow exhibited immune complex formation in the kidney. This result indicates that the Pol β-Y265C protein must be expressed in cells other than those of the hematopoietic compartment in order for immune complex formation to be observed in the kidneys of the mice. One study using chromatography combined with mass spectrometry has demonstrated the presence of oxidized DNA in the kidneys of mice [15]. The BER pathway is a major homeostatic mechanism to maintain low levels of oxidative DNA damage in tissues and a key protein in that pathway is Pol β. The Pol β-Y265C protein has extremely low catalytic activity, leading to the accumulation of BER intermediates and these intermediates can result in cell death [16]. Dying cells in the kidneys of *POLB*^Y265C/C^ mice could provide antigens that are recognized by circulating IgG, leading to immune complex formation. Alternatively, expression of the defective Pol β-Y265C protein in other tissues of the *POLB*^Y265C/C^ mice could result in aberrant BER leading to an increase in the levels of dying cells in the mice that eventually result in immune complex localization in the kidney. Regardless, our results indicate that hematopoietic cells transplanted from *POLB*^Y265C/C^ mice are sufficient for development of renal disease.

### Higher levels of activated B cells in the spleens of Pep Boys reconstituted from the bone marrow of the *POLB*^Y265C/C^ mice

**Fig 3:**
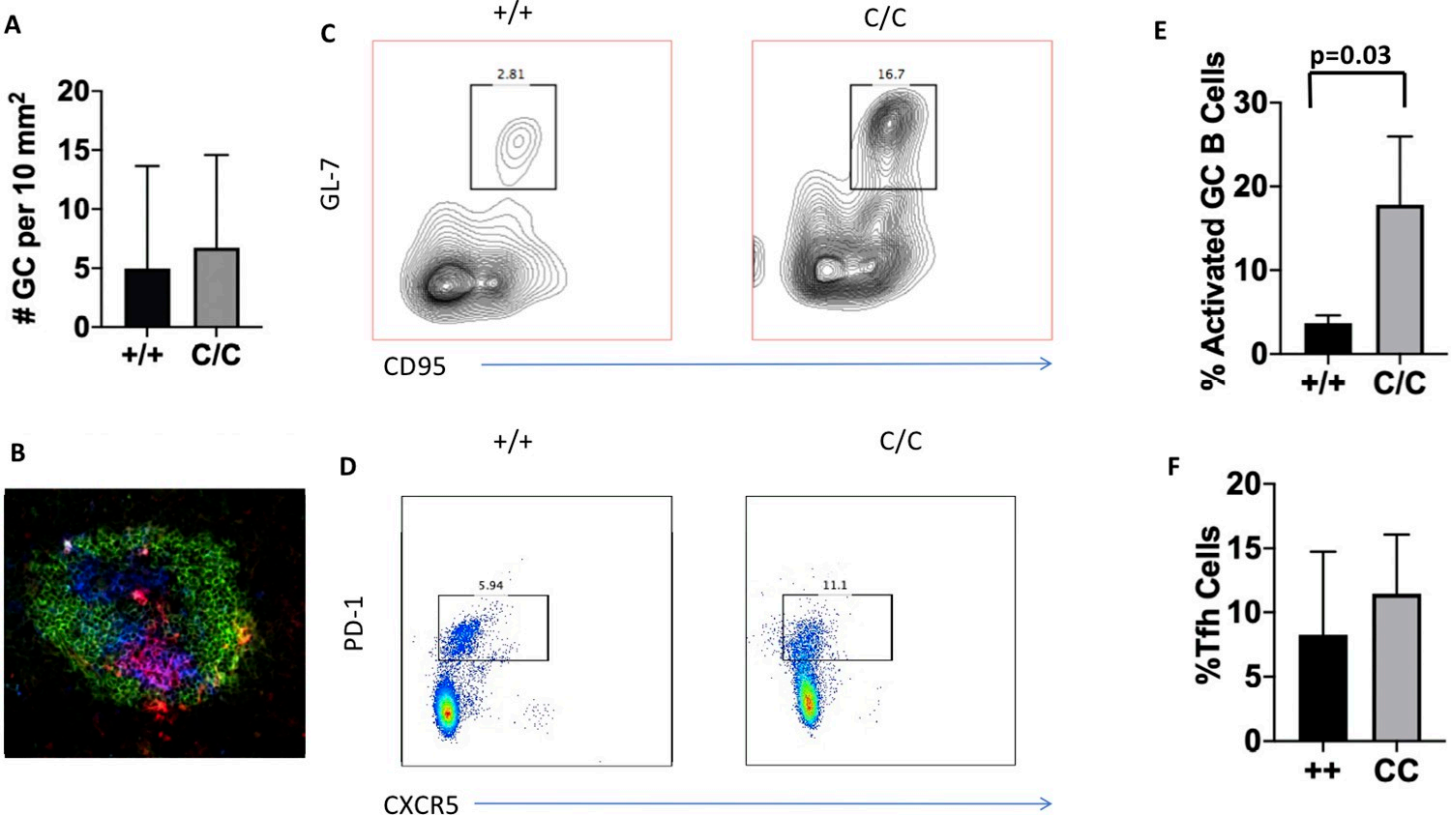
Increased levels of activated B cells in spleens of the *POLB*^Y265C/C^ mice. A. The numbers of germinal centers are similar in the *POLB*^+/+^ mice and *POLB*^Y265C/C^ mice. B. Representative example of a germinal center. Activated B cells stained with antisera raised against lectin PNA are green; T-cells are stained with antisera raised against CD4 in blue; cells staining for IgD-PE are red. C. Representative FACs plot of GL7+CD95+ cells (activated B cells). D. Representative FACs plot of PD-1+CXCR5+ Tfh cells. E, F. Quantification of activated B cells and Tfh cells, respectively. T-tests were used to determine if the means of each of the two groups were significantly different from each other.

Given that numbers of germinal centers (GCs) were increased in the *POLB*^Y265C/C^ compared to the *POLB*^+/+^ mice in our previous study [3]. we quantified the numbers of germinal centers in Pep Boy recipients of bone marrow from either the *POLB*^Y265C/C^ or *POLB*^+/+^ mice as we describe in our study. No differences were observed (Fig 3A and 3B). We also quantified the levels of activated B cells and follicular helper T cells (Tfh) in the spleens of these mice. The recipients of the *POLB*^Y265C/C^ bone marrow had significantly increased levels of activated B cells in their spleens compared to the *POLB* ^+/+^ recipients (Fig 3C and 3E). Although the recipients of the *POLB*^Y265C/C^ bone marrow trended towards increased levels of Tfh cells compared to recipients of *POLB*^+/+^ bone marrow, the differences were not significant (Fig 3D and 3F).

### Skin lesions are not present in recipients of *POLB*^Y265C/C^ bone marrow

Skin lesions were observed in significantly increased percentages of the *POLB*^Y265C/C^ versus *POLB*^+/+^ mice as a function of age [3]. In contrast, skin lesions were not observed in recipients transplanted with bone marrow from the *POLB*^Y265C/C^ mice. This result indicates that skin lesions may form as a result of expression of the Y265C protein in the skin.

At 9–12 months of age, approximately 40% of the *POLB*^Y265C/C^ mice develop skin lesions with lupus-like characteristics [3]. The skin lesions we observed in these mice are characterized by acanthosis, hyperkeratosis, follicular plugging, lymphocytic inflammation, and interface dermatitis, resembling discoid lupus in humans. In contrast, none of the mice engrafted with bone marrow from the *POLB*^Y265C/C^ mice develop skin lesions. This indicates that skin lesions at least in part result from expression of the Pol β-Y265C protein in the skin and that engraftment with hematopoietic cells is not sufficient for the development of skin lesions. Reactive oxygen species are produced by the mitochondria of both keratinocytes and fibroblasts in the skin (for a review see [17]). These ROS could induce oxidative DNA damage that is repaired by the BER pathway. In the presence of the catalytically slow Pol β-Y265C, we propose that BER intermediates, including single nucleotide gaps and DNA breaks, accumulate in the skin, leading to inflammation. Infiltration of immune cells into the skin could also increase ROS levels, resulting in DNA damage and aberrant repair in the presence of Pol β-Y265C. Alternatively, Pol β is suggested to be present in mitochondria [18, 19] and the deficient repair of mitochondrial DNA by Pol β-Y265C could increase ROS in the skin, perhaps resulting in skin lesions.

In summary, we show that transplantation of bone marrow from the *POLB*^Y265C/C^ mice, but not wild-type controls, results in many of the characteristics of lupus including high levels of ANA and renal disease. We conclude that the hematopoietic compartment is sufficient for development of lupus in our mouse model, and that the underlying mechanism is likely associated with aberrant DNA repair during the development of the immune repertoire. Interestingly, the renal disease observed in the recipients of the *POLB*^Y265C/C^ bone marrow was not accompanied by immune complex formation in the majority of the mice we characterized. In addition, the hematopoietic stem cells were not sufficient for the development of cutaneous disease. These results indicate that expression of the Y265C protein in the kidney and skin plays a critical role in organ-specific manifestation of lupus.

## Supporting information

S1 Fig Gating strategy for activated B cells. (TIFF)

S2 Fig Gating strategy for follicular helper T cells. (TIFF)

S1 Data (XLSX)
