# Supplementary figures and images for: The hematopoietic compartment is sufficient for lupus development resulting from the POLB-Y265C mutation
Source: PLoS One. 2022 Apr 29;17(4):e0267913. doi: 10.1371/journal.pone.0267913 (PMC9053796; doi:10.1371/journal.pone.0267913)

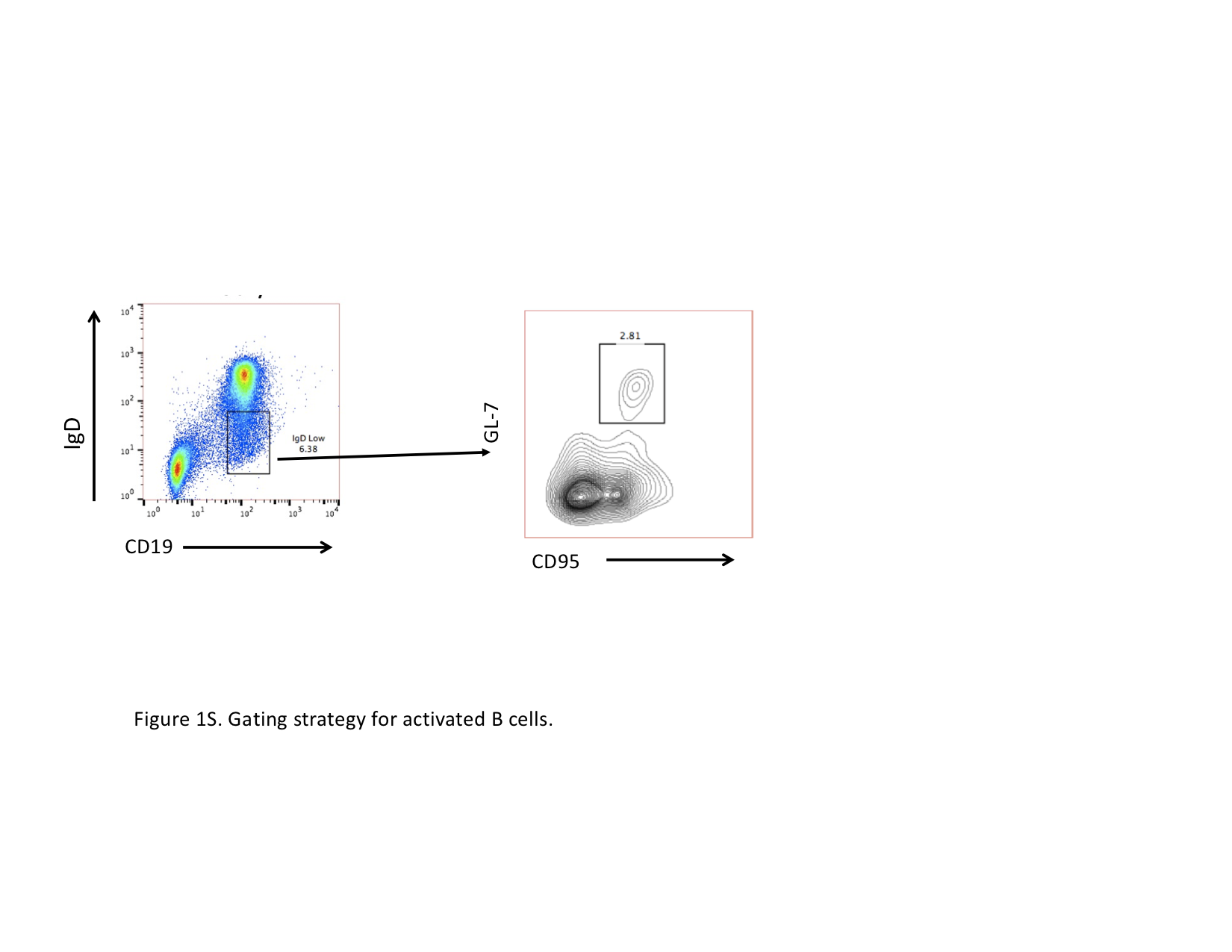

Supplement: S1 Fig — (TIFF) [file pone.0267913.s001.tiff]

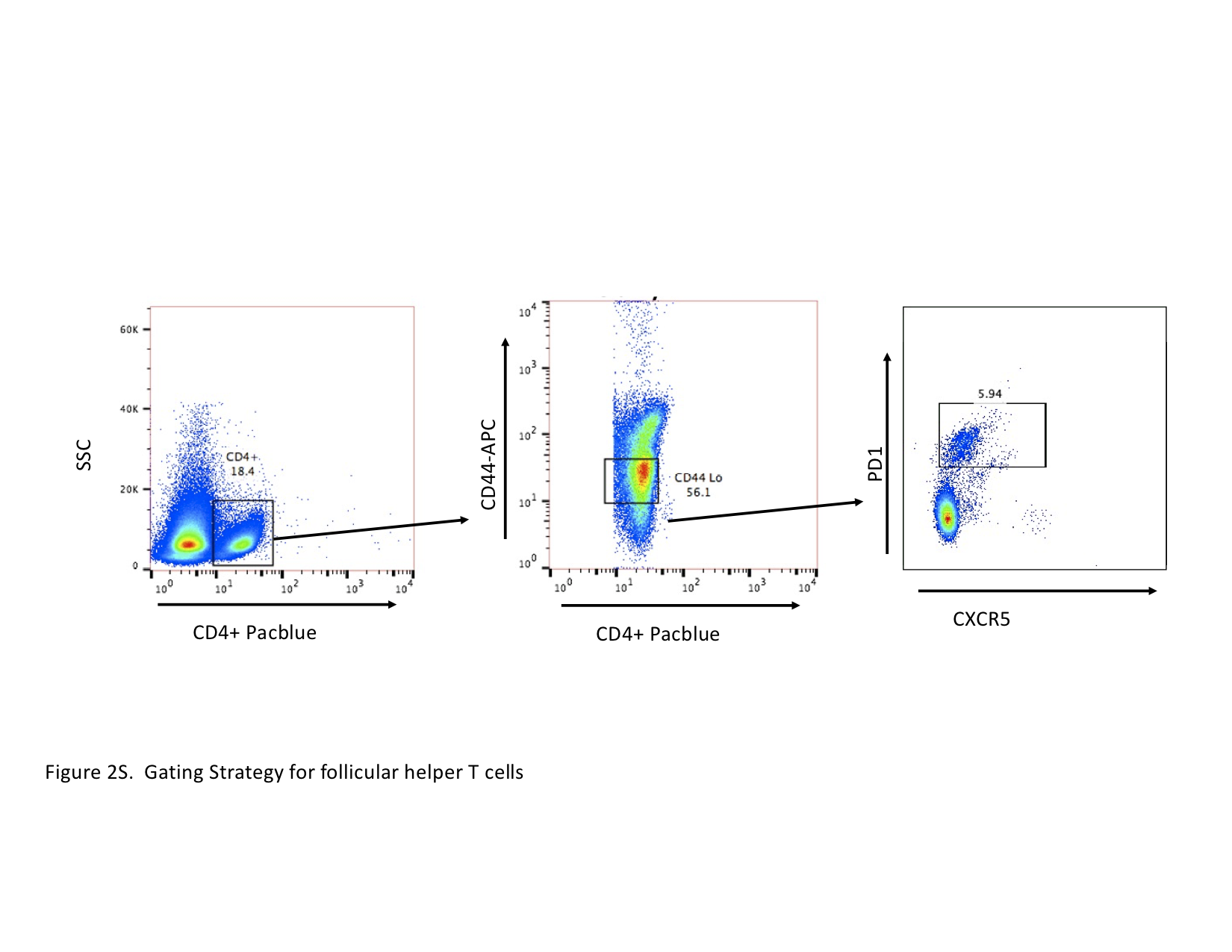

Supplement: S2 Fig — (TIFF) [file pone.0267913.s002.tiff]
